# Autologous Lens Capsule Flap Transplantation for Persistent Macular Holes

**DOI:** 10.1155/2021/8148792

**Published:** 2021-02-27

**Authors:** Sławomir Cisiecki, Karolina Bonińska, Maciej Bednarski

**Affiliations:** Centrum Medyczne “Julianów”, 91-321 Łódź, ul, Żeglarska 4, Poland

## Abstract

**Purpose:**

To analyze the anatomical and functional outcomes after autologous lens capsule transplantation in patients with persistent macular hole.

**Methods:**

This is a retrospective observational study of five eyes of five patients treated with vitrectomy and autologous lens capsular flap transplantation. Complete ophthalmic examination was performed preoperatively and seven days and 1, 3, 6, 12, and 18 months after surgery.

**Results:**

Successful macular hole closure was achieved in all patients. The mean minimum macular hole diameter before the surgery was 666.8 *µ*m, and the mean basal diameter was 1086.4 *µ*m. The mean visual acuity before lens capsular flap transplantation was 20/200, while after surgery, it was 20/125.

**Conclusions:**

Autologous lens capsular flap transplantation is a potential alternative treatment for patients with large persistent macular holes after other operative techniques have failed.

## 1. Introduction

*Pars plana* vitrectomy with internal limiting membrane (ILM) peeling has been a standard procedure in macular hole (MH) treatment for over a decade [1]. On average, hole closure is achieved in 88–100% of the cases [2, 3].

Operative failures might occur with large, persistent holes, exceeding 400 *µ*m in diameter. Such failures have encouraged surgeons to search for modifications to the conventional technique, often using autologous tissue as a scaffolding for cell migration from the surrounding retina, including inverted ILM flap, autologous ILM graft from the surrounding retina, autologous retina, autologous blood application, and lens capsule [4–10].

**Figure 1:**
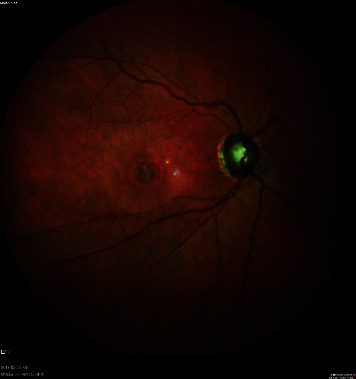
A macular hole closed surgically with a lens capsular flap.

**Figure 2:**
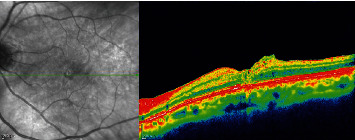
Spectral-domain optical coherence tomography (SD-OCT) image of a macular hole closed surgically with a lens capsule.

One modified technique involves transplanting material from the anterior or posterior lens capsule onto the MH [5, 7, 8] (Figures 1 and 2). This technique proved effective in persistent holes after extensive ILM peeling and is the main topic of this article.

## 2. Materials and Methods

This report concerns five eyes with persistent full-thickness MH in five female patients (average age 60.6 years; range 43–73 years). All patients were treated unsuccessfully by 20-gauge vitrectomy with ILM peeling and were scheduled for modified lens capsular flap transplantation. Written consent for the surgical procedure was obtained from all patients.

A silicone oil tamponade with its subsequent removal was used in four patients, whereas the remaining patient received an air tamponade.

All patients underwent a full preoperative ophthalmic examination. The slit-lamp biomicroscopy was performed after dilating the pupils with 1% tropicamide solution.

SD-OCT examinations were performed using the SPECTRALIS HRA + OCT system (Heidelberg Engineering, Germany). The analysis included B-scans, performed 12 mm from the center at the fovea level. Preoperatively, the minimum hole diameter and the base diameter at the retinal pigment epithelium level were measured.

Transplantation of a flap harvested from the posterior lens capsule was performed in four pseudophakic patients, and phacovitrectomy was done in one patient, using material from the anterior lens capsule of the same eye to close the hole.

All patients underwent surgery by the same surgeon (S. C.). Postoperative examinations took place one day and 1, 3, 6, 12, and 18 months after the surgery. The patients underwent a full ophthalmic examination during the follow-up visits that included SD-OCT, similar to the preoperative schedule.

### 2.1. Surgical Technique

#### 2.1.1. Step I

All surgeries were performed following periocular anesthesia and with a 20-gauge system.

In an eye with an immature cataract, surgery began with its removal and the implantation of an artificial lens in the posterior capsule. The anterior capsule was stained with trypan blue for better visualization, followed by capsulorhexis with a cystotome needle.

In the four pseudophakic patients, the surgery began with posterior lens capsule staining by direct injection of trypan blue, followed by posterior rhexis with an ILM forceps.

During the operation, Membrane Blue Dual® (Dorc, Rotterdam, the Netherlands) was used to stain the remaining ILM around the MH to exclude the possibility of performing an autologous free ILM flap. The dye was rinsed out after 1 min.

The size of the capsular flap harvested in each case was larger than the diameter of the MH.

#### 2.1.2. Step 2

After harvesting the lens capsular flap, it was placed gently with the outer surface over the MH. The eye was filled completely with perfluorocarbon liquid (PFCL) to decrease the risk of the displacement by residual fluids.

#### 2.1.3. Step 3

In four cases, a direct exchange of PFCL with 1000 Cs silicone oil was performed to ensure that the unattached posterior capsular flap was not lost. In the one case where an anterior capsular flap was employed, PFCL/air exchange was performed and the patient was informed about the necessity of maintaining a face-down position for three days after the surgery.

## 3. Results

### 3.1. Overall Results

**Figure 3:**
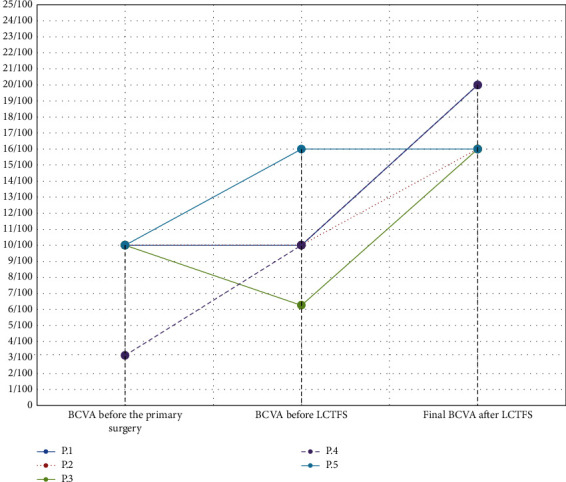
Changes in the BCVA from baseline to 18 months after the surgery.

Successful MH closure was achieved in all patients. The silicone oil endotamponade was removed after one week. The mean minimum MH diameter before surgery was 666.8 *µ*m, and the mean basal diameter was 1086.4 *µ*m. The mean best-corrected visual acuity improved significantly (Figure 3).

Before the lens capsular flap transplantation, the mean preoperative visual acuity was 20/250, while it was 20/125 after surgery. Detailed data are presented in Table 1.

### 3.2. Case Examples

**Figure 4:**
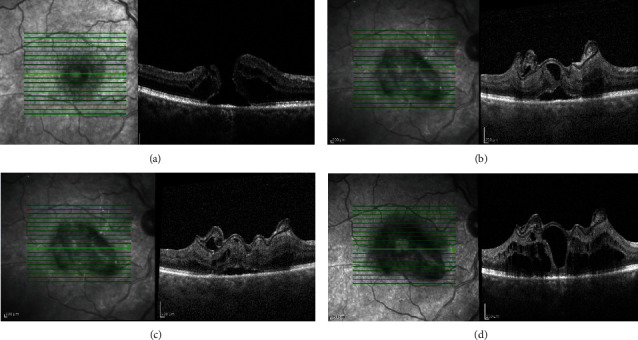
Macular hole closure over time after anterior lens capsular flap transplantation. Vertical SD-OCT scans: (a) baseline, (b) one month after surgery, (c) six months postoperatively, and (d) 12 months postoperatively. Scale bar = 200 *µ*m.

**Figure 5:**
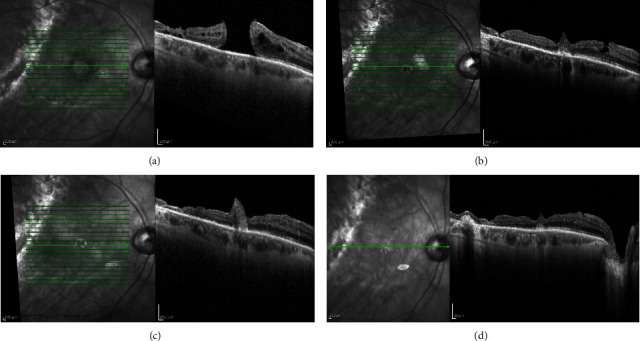
Macular hole closure over time after posterior lens capsular flap transplantation. Vertical SD-OCT scans: (a) baseline, (b) one month after surgery, (c) six months postoperatively, and (d) 12 months postoperatively. Scale bar = 200 *µ*m.

The postoperative scans showed closure of the hole with gradual restoration of the retinal layers. Figures 4(a)–4(d) and Figures 5(a)–5(d) show cases of anterior and posterior lens capsular flap transplantation, respectively.

## 4. Discussion

Considering the variety of operative techniques available for MH treatment, it is possible to reduce the number of persistent holes. In the traditional, conventional, operative technique of vitrectomy with ILM peeling, the gas promotes for cell migration, allowing the closure of the hole and separation of the pigment epithelium from the liquid. In the inverted ILM flap technique or when transplanting ILM from the margin of the retina, it is for the ILM flap to bridge the hole, while the air serves as an additional stabilizer [8, 10].

Vogt et al. found positive immunoreactivity of macroglia and microglia cells in the transplanted posterior lens capsule (PLC) [11]. Michalewska et al. suggested that if a segment of the peeled-off ILM is left attached, it might provoke gliosis inside the retina and ILM surface. Comparable immunostaining of the PLC material and ILM may suggest that the MH closure mechanism in the inverted ILM flap technique and PLC transplantation is similar [11].

Chen and Yang hypothesized that the lens capsule, like the basement membrane, facilitates bridging the retinal tissue above the hole (like an ILM flap) [5]. The lens capsule has a pliable consistency. Moreover, it has a higher density than the ILM, making it easier to settle on the retinal surface and be directed to the designated place [5]. The use of lens capsular flap transplantation requires some technical aspects to be discussed. First, the graft size should be more than 1 mm larger than the MH diameter, even in cases of large hole. A very important tip is to close the infusion line to decrease fluid turbulence and to avoid transplant displacement.

Another interesting issue worth clarifying is the graft orientation. We tried to place lens capsular transplants with the outer surface onto the hole, as it is smoother than the inner one and contains no cells. However, it might be difficult to differentiate between the sides after the manipulations under the PFCL.

There are certain differences between using the anterior and posterior capsules during surgery. The anterior capsule is thicker and when used in conjunction with PFCL adheres firmly to the retinal surface. With the anterior capsule implantation in mind, it was decided to use an air endotamponade in this patient (no. 5 in Table 1). In this case, we observed intraretinal edema with almost complete external limiting membrane restoration, ellipsoid zone, and clearly seen retinal pigment epithelium. First, the anterior capsule was not very smooth, and it contained cells that promoted cell migration; second, we may have unintentionally placed the transplant in an upside-down orientation during manipulations under the PFCL.

In the four eyes in which a posterior capsular autograft was performed, silicone oil was used for endotamponade to prevent dislocation of the flap. The silicone oil was removed after one week. We observed flap incorporation into the retinal tissue during follow-up SD-OCT scans of two patients (Figure 2; patient nos. 1 and 2). The foveal contour was defined as a U-shape closure with small irregularity. We observed a type of plug in the other two patients (Figures 4(b)–4(d); patient nos. 3 and 4) which probably confirmed that the flap, like the basement membrane, could bridge the hole and represent a membranous noncellular tissue. We know that this might influence BCVA but the exact impact is unknown [12].

The final visual acuity improved over the preoperative state, but it remained relatively poor. It should be mentioned that, unlike our patients, the holes would remain open after performing a classical vitrectomy with ILM peeling [10].

## 5. Conclusions

Lens capsular flap transplantation gives hope to patients with large MH, in whom other operative techniques have failed. Therefore, it seems reasonable to retain the posterior capsule during combined surgeries in case it will be needed for other indications.

Our work was based on a small case series. Even though positive postoperative results were achieved, it is necessary to repeat the study with a larger cohort.

## Figures and Tables

**Table 1:** Patients' data.

Patient no.	Sex/age	MH etiology	BCVA before the primary surgery (Snellen)	BCVA before lens capsule transplant surgery (Snellen)	Final BCVA post-op. (Snellen)	MH (min/basal) before the primary surgery (Snellen)	MH (min/basal) before LCTFS (*µ*m)	Transplant	Previous surgical procedures/primary pathology
**1**	F/43	Primary	0, 1 20/200	0, 1 20/200	0, 2 20/100	936/1705	951/1396	Posterior capsule	(1) Phacovitrectomy + sil.oil/MH
**2**	F/68	Primary	0, 1 20/200	0, 1 20/200	0, 16 20/125	605/1020	603/1017	Posterior capsule	(1) Vitrectomy + 100%air/MH
**3**	F/60	Primary	0, 1 20/200	0, 06 20/320	0, 16 20/125	905/1508	1065/1611	Posterior capsule	(1) Vitrectomy + ILM peeling + sil. oil/MH with retinal detachment (2) Sil. oil removal/persistent MH
**4**	F/59	Secondary (after blunt trauma)	0, 03 20/640	0, 1 20/200	0, 2 20/100	582/647	445/600	Posterior capsule	(1) Phacovitrectomy + 100% air/MH
**5**	F/73	Primary	0, 1 20/200	0, 16 20/125	0, 16 20/125	389/1150	270/808	Anterior capsule	(1) Vitrectomy + 100% air/MH

F: female; BCVA: best-corrected visual acuity; MH: macular hole; LCTFS: lens capsular flap transplantation surgery; No.: number; sil. ol: silicone oil.

## Data Availability

The patient data used to support the findings of this study are included within the article.

## Additional Points

Partial results from this study (12-month follow-up) were presented at the MaculArt 2019 Meeting, June 2019, and EVRS 2019, May 2019.
